# Adaptation, Academic Performance and Support: Students with and without Disabilities and Future Considerations for Counselling Psychology

**DOI:** 10.3390/bs13100862

**Published:** 2023-10-20

**Authors:** Panagiotis Parpottas, Yianna Christofi, Ioanna Ioannou

**Affiliations:** 1 Department of Social and Behavioral Sciences, School of Humanities, Social and Education Sciences, European University Cyprus, 2404 Engomi, Cyprus; 2 Committee for Students with Special Educational Needs, European University Cyprus, 2404 Engomi, Cyprus; efeea@euc.ac.cy (Y.C.); i.ioannou@euc.ac.cy (I.I.)

**Keywords:** university students, disabilities, adaptation, performance, academic support, counselling psychology

## Abstract

The transition to university is a process that presents young adults with several challenges in adaptation, especially students with disabilities. The current study investigated the differences in adaptation and academic performance between students with and without disabilities and further examined these differences among students with disabilities. Additionally, we explored the role of academic support for students with disabilities’ adaptation and academic performance, as well as their perceptions of a proposed specialised package of counselling psychology interventions. The sample consisted of 127 students with disabilities and 127 without disabilities, aged 18–24 years. Results revealed that students with disabilities reported a lower GPA and adaptation than students without disabilities. Additionally, differences among students with disabilities were observed only in terms of GPA, according to their disability type, existence of comorbidity and type of exam accommodations. Interestingly, no differences were found in GPA or adaptation between students with disabilities who utilised psychological therapy and those who did not. Finally, adaptation scores, but not GPA, were higher for students with disabilities who were positive in receiving a specialised package of counselling psychology interventions. The findings are discussed in relation to the existing literature and future considerations of counselling psychology’s role in support of students with disabilities.

## 1. Introduction

The growing number of students entering higher education (HE) has created the need to further understand young adults’ university experience. This need became more imperative since the transition to university coincides with a developmental phase during which young adults leave the family home and face several challenges in their ability to adapt to a new environment [1]. Adaptation to university is a multifaceted process that refers to students’ ability to adjust to a new environment and consists of ways of interaction with the academic setting and coping with the transition at an academic, social, personal–emotional and institutional level [2]. It is generally accepted that the majority of students can successfully manage the personal and academic demands of their new life (i.e., experience less social and mental health problems and achieve decent grades), while others encounter several difficulties in their adaptation to university [3,4].

The most considerable body of research has primarily investigated how the general student population adapts to university, while the experience of students considered at risk, such as students with disabilities (SWD), is often left outside the research scope. As a result, SWD are underrepresented in the literature. Additionally, only a small number of studies explored adaptation and academic performance differences between the general student population and SWD. Furthermore, the literature provides us with complex findings on how the provided support can help SWD achieve a smooth adaptation and academic performance, and more importantly, it highlights the absence of counselling psychology from SWD’s support. Therefore, the aim of this empirical study was triple: firstly, to identify adaptation and academic performance differences between university students with and without disabilities, secondly to further explore these differences among SWD, and, finally, to investigate the role of the existing support system on SWD’s academic experience, as well as their perceptions on a proposed package of counselling psychology interventions.

### 1.1. Differences between Students with and without Disabilities

The most common disabilities reported in HE are vision, hearing, speech, mobility and cognitive impairments, learning, mental health and physical disabilities, autism, ADHD, and other medical problems or chronic health conditions [5,6,7]. The literature focused on SWD has revealed a clear connection between these disabilities and academic difficulties [8], but one could argue that although the transition to university is challenging for many young adults, the difficulties faced by SWD may not be similar to those of the general student population.

A number of studies that compared students with and without disabilities revealed differences in academic achievement, social integration [9], psycho-emotional status [10], graduation rates [11], and professional opportunities after graduation [12], with unfavorable outcomes for SWD. More specifically, Lombardi et al. [13] found that compared to their non-disabled peers, SWD face various challenges and obstacles in their academic pursuits. These include poor academic performance, difficulties with learning facilities or barriers in accessing educational resources and support. Another study has reported that SWD often experience more academic difficulties and lower grades compared to their fellow students without disabilities due to cognitive and processing challenges [14]. Furthermore, when compared to students without disabilities, SWD have lower self-esteem, which is associated with negative academic performance [15]. Finally, Blanco et al. [16] found that students with SWD had a higher risk of experiencing mental health problems such as depression and anxiety, possibly due to additional stress and challenges associated with their disability.

Looking at these differences, it is evident that SWD face more difficulties than students without disabilities, leading us to postulate that these difficulties can negatively affect their adaptation and performance. As a matter of fact, Adams and Proctor [17] investigated the relationship between students with and without disabilities’ overall college adjustment, social adjustment, institutional attachment, and semester GPA and found that students without disabilities scored higher in these areas. This study’s results highlighted that SWD do not adapt well and hence are more at risk in terms of having unsuccessful social relationships, not feeling committed to their institution and having lower academic performance. Respectively, Lipka et al. [18] found that the adjustment of SWD compared to non-disabled students was less successful and suggested that SWD’s difficulties in all elements of adjustment (academic, social, personal–emotional and institutional) were associated with specific patterns according to their disability. In a very recent study [19], it was reported that SWD showed a marked difference in academic performance compared to their peers without disabilities, with a significantly lower GPA and a higher probability of failing courses in their first year of study. Therefore, considering all findings, it seems evident that the existence of a disability hinders SWD’s attempt to successfully adjust in higher education. Difficulties in engagement with the academic setting and not being able to develop the coping mechanisms to withstand the developmental changes in the transition to university can create several other strains in academic performance, as well as in the social and emotional aspects of a student’s life. Thus, it is imperative to understand the process of adaptation for students at risk, such as SWD, in order to consider how specialised support can further assist them to have a smoother adaptation during their studies.

### 1.2. Support for SWD

To tackle the challenges posed by disability, international standards and inclusion policies, national and local legislations are set to ensure that SWD will receive support services and programs designed to address their needs and help them overcome the academic barriers in HE [3,20,21,22,23,24,25,26,27,28,29]. These support services include tutoring and supplemental instruction [30], mentoring, educational advising, academic coaching and career advising, financial support, social interventions and counselling [17,31]. However, academic accommodations appear to be the most frequently reported type of support provided by universities internationally and consist, among others, of instructional adjustments, examination time extensions, simplification and clarification of reading materials and exams, guidance in organisation and planning of studying and provision of technological tools [3,17,19,27,32,33,34,35]. In addition, research has clearly shown that therapeutic counselling can be beneficial for students’ mental health and academic careers and that its effectiveness can be maintained over time [36]. Moreover, previous studies highlight the significant effect of counselling on academic performance, transitional issues and retention [37]. Nevertheless, only a scant number of studies have explored the benefits of therapeutic counselling for SWD.

Therefore, even though there is an array of significant services available to support SWD, the current study will specifically focus on academic accommodations and therapeutic counselling for three main reasons. The first is that although academic accommodations have received considerable attention in the literature and appear to be the primary means of support for SWD, the available studies which have examined their actual effect on SWD’s performance or adaptation provided us with mixed findings. Secondly, although therapeutic counselling is highly considered an effective mean of support for university students, only a few studies have examined their actual effect on SWD’s performance and adaptation. Finally, the lack of such research in Cyprus motivates us to explore and understand if the two primary services that we offer to SWD, which are academic accommodations and therapeutic counselling, can help students and how these services could become more effective in the future.

#### 1.2.1. Effectiveness of Academic Accommodations on SWD’s Adaptation and Academic Performance

Even though SWD report satisfaction with their accommodations and despite the fact that accommodations enhance SWD’s engagement and participation in HE [3,38], research on the effectiveness of academic accommodations on SWD’s adaptation and performance provides us with mixed findings.

The first stream of studies presents both a positive and a conditionally positive effect of academic accommodations on GPA and adaptation. For example, a few studies have indicated that access to academic support services was related to higher GPA and graduation rates [39,40,41] and that both exams and class accommodations had a positive effect on overall GPA [42]. Additionally, some researchers have found a conditionally positive association between accommodations, GPA and adaptation. Specifically, Dong and Lucas [43] reported that students with cognitive and psychological disabilities who used accommodations presented an improved academic performance in the third semester of study than students with physical disabilities or no disabilities. Similarly, Trammell [44] found that students with attention deficit disorder (ADD) and students with learning disabilities (LD) and ADD had significantly improved grades when using academic accommodations, whereas students with only LD had a drop in their grades. Finally, Mamiseishvili and Koch [25] found a significant association between academic accommodations and first-to-second-year persistence of SWD. However, these associations declined in significance when controlling for some in-college characteristics.

The second stream of studies presents a different and even more complex picture of the effect of academic accommodations on GPA and adaptation. For instance, Yuval and colleagues [45] found that accommodations had positive effects on students’ learning, retention and completion rates but not in improving their GPA. Similarly, Moon et al. [46] found that inclusive practices had a positive effect on retention and completion rate but not GPA. Moreover, another study [47] reported that although inclusive practices help SWD adapt and perform better, some practices had a negative effect on these outcomes. For example, course modifications which were earned in high school and continued in college had a negative effect on adaptation and academic performance during the first term, and students were not able to adjust to the reality of college. Additionally, after a year in college, the use of accessible course materials did not help students adjust to the new environment of college; on the contrary, they had to readjust for each course in each term. In the study of Adams and Proctor [17], only self-advocacy skills (e.g., self-knowledge, knowledge of rights, communication and leadership skills) and SWD’s perception of the visibility of their disability to other people predicted their adjustment. In addition, overall GPA scores were not significantly different between students with and without disabilities, with the authors presuming that academic accommodations were successful in assisting SWD’s performance. Therefore, this study concluded that although academic accommodations may assist SWD to perform equally to students without disabilities, other means of support provided by disability and counselling centres may be more critical in helping SWD build better social and emotional skills, which will ultimately help them adapt to university. Additionally, Parsons and her colleagues [19] found that the number of the provided academic accommodations was not significantly associated with GPA or with failed/dropped courses and also that notetaking, calculator use and other exam formats were associated with poorer GPA. Finally, a study suggested that a higher GPA was associated with lower adaptation to college for SWD [48], and this was the only study which suggested that academic performance may not be an accurate indicator of academic adaptation for SWD. Although this result is quite unique, it stresses that SWD may still be at risk even if they perform well. Hence, campus-disability services should focus on utilising alternative methods to assess factors which can hinder SWD’s university adaptation and offer interventions that may not primarily relate to academic performance; for example, mentoring, psychoeducation and therapeutic counselling could be some options based on previous research [35,36].

#### 1.2.2. Effectiveness of Counselling on SWD’s Adaptation and Performance

As we already know, the transition to university is associated with increased distress, which is closely related to higher drop-out rates [49,50,51]. Conversely, a positive transition can enable students to have a better experience and even increased academic success [52]. Therefore, by offering counselling services, universities aim to strengthen the support mechanisms which can help their students transition to university and, at the same time, aid them in overcoming challenges during their studies. Due to the very small number of relevant studies on the effect of counselling on SWD’s adaptation and academic performance, we firstly review the literature on the general student population in order to use it as a baseline and then present the three studies we found specifically for SWD.

Generally, it can be understood that campus counselling/psychotherapy plays an important role in students’ overall positive experience [53]. More specifically, it contributes to the decrease in psychological symptoms and in the improvement of students’ wellbeing [54,55,56,57]. Eleven studies report statistically significant findings of counselling’s positive effect on university retention [37,58,59,60], academic performance, outcome and GPA [61,62,63,64], as well as on university adaptation [65,66,67]. From those 11 studies, two studies presented specific conditions in their results’ significance, and two other studies did not find any relationship between counselling and GPA. More specifically, Cholewa and Rmaswami [61] found that although counselling had a positive effect on GPA, only 3–4 h of counselling produced this positive effect on the first semester of freshmen students and this effect was no longer significant in the following semester. The second study [37], which reported a positive effect of counselling on student attrition, has also indicated that counselling had a negative association with GPA (result with low statistical effect). Nevertheless, this negative association changed to significantly positive for students who attended both individual (IC) and group counselling (GC), compared to students who received either only IC or only GC or any other support service. Lastly, only one study reported that counselling was unrelated to academic performance [68], and another study did not find any significant differences in GPA and degree completion between users and non-users of counselling but found that counselling-users graduated at a significantly lower rate compared with non-users [69].

The results from studies carried out in the general student population provide us with a clear view of the effectiveness of counselling/therapy on students’ adaptation and academic performance. However, the scant number of studies specifically focused on SWD provide us with a complex picture. The first study reported that counselling helped students who faced academic problems improve their academic work, their retention and overall university experience. Finally, these students showed reliable clinical change in symptomatology compared to students who did not face any academic problems [70]. A later study [71] found that compared to students without disabilities, SWD significantly dropped out of therapy and were more likely to be referred to external sources of help, suggesting that SWD could not gain similar positive counselling outcomes as other students. Finally, a more recent study [72], which compared SWD, students with psychiatric problems and students without disabilities, reported that therapy significantly reduced all three groups’ overall and academic distress, but when controlling for time of therapy and group difficulties, it was indicated that over the course of therapy, SWD experienced significantly less change in overall and academic distress than students without disabilities. Considering all the above, we conclude that although counselling may be generally promising for students, there is little evidence to help us understand its effectiveness for SWD. Moreover, another neglected area is the work of counselling psychologists with disabilities [73], and scholars call for further research [74,75].

### 1.3. Aim and Research Questions of the Current Study

Based on the reviewed literature, it is clear that students with and without disabilities have significant differences in adaptation and academic performance and that both academic and therapeutic support may be somehow helpful for SWD under specific conditions. Therefore, our study’s aim was to identify adaptation and academic performance differences between university students with and without disabilities and to further explore these differences among SWD. In addition, we wanted to understand the role of the existing support system on SWD’s academic experience and to explore their perceptions of a proposed package of counselling psychology interventions. This could help us investigate the prospect of offering SWD a supplementary means of support from a counselling psychology perspective and design future studies which could test such interventions and ultimately contribute to the field of supporting SWD. This study was designed to answer the following questions:

1. Are there any differences in GPA and adaptation between students with and without disabilities?
2. Do SWD have differences in GPA and adaptation according to: (a) the type of their disability and (b) the existence of disability comorbidity?
3. Do SWD have differences in GPA and adaptation according to their type of academic accommodations?
4. Does the existence of psychological therapy differentiate SWD in GPA and adaptation?
5. Are there any differences in SWD’s GPA and adaptation scores according to their perceptions of a proposed specialised package of counselling psychology interventions aiming to enhance their academic experience?

## 2. Materials and Methods

### 2.1. Participants

Students at a leading private university in Cyprus were asked to participate in the survey three weeks into the Spring semester. The sample of the study consisted of 254 students, 127 were SWD and 127 without disabilities, 112 men and 142 women aged 18–24 with an average age of 21.02 years (SD = 1.90). A purposeful sampling procedure [76] was employed to increase the probability of a representative sample of SWD. Additionally, SWD were only reached through the Committee for Students with Special Educational Needs (CSSEN). No attempt was made to include SWD not registered with this service. The response rate was 59% for SWD (a total of 215 registered students).

### 2.2. Measures

The demographic characteristics of the sample were collected with simple questions regarding gender, age, nationality, residence status, level of study, program of study, year of study, GPA, disability type, comorbidity, type of provided academic accommodations and information about psychological therapy. Finally, a single question assessed SWD’s beliefs about whether extra support could enhance their academic experience (consisted of the following interventions: psychoeducation of disability, mindfulness, social-emotional learning techniques, working with acceptance, self-stigma, attributional styles and self-determination).

Students’ adaptation was assessed by the Greek version of the College Adaptation Questionnaire (CAQ) [77]. This questionnaire was originally developed by Crombag [78], contains 18 items and provides a general score of the university adaptation, indicating whether a student feels satisfied with the university and their program of study and with their socialisation at the university. Participants responded to each item on a Likert scale ranging from 1 (it doesn’t apply to me at all) to 7 (it applies to me very much), with higher scores representing better general adjustment. The questionnaire had good internal consistency with Cronbach’s alpha = 0.89 in both English and Greek versions [79,80]. Additionally, the construct validity of the Greek version was confirmed by the Student Adaptation to College Questionnaire with r = 0.73 [80]. The Cronbach’s alpha of the CAQ for this study was 0.83.

### 2.3. Design and Procedure

To investigate our research questions, a between-participants design was utilised. To examine the first research question, the independent variable consisted of two levels: SWD and students without disabilities, and the dependent variables were GPA and adaptation. Finally, to test all other research questions between the sample of SWD, GPA and adaptation were our dependent variables, and the six independent variables were disability type (consisted of six levels), comorbidity (consisted of two levels), teaching accommodations (consisted of two levels), exam accommodations (consisted of three levels), psychological therapy (consisted of three levels) and extra support (consisted of two levels).

The study received ethics clearance and approval from the National Bioethics Committee of Cyprus. The committee deemed that its procedures were in accordance with requirements for research involving human participants. The study also abided by the operating regulations of the CSSEN, such as confidentiality and data protection.

The recruitment of participants took place electronically and with posted announcements around the university campus. CSSEN’s support officers sent their registered students an email with the study’s description and the option to either complete the questionnaires online via a Microsoft office forms link or to complete the questionnaire at the CSSEN reception and, after its completion, to submit it by placing it in a designated box. Additionally, invitation emails for research participation were sent to the six schools of the university, requesting them to forward the message to their students. Finally, the same research description-invitation was posted on announcement boards at the university campus. The majority of participants (74.8%) chose to complete the questionnaire online, while 25.2% completed it in hard copy. Prior to entering the study, all interested students had sufficient time to read the description and purpose of the research and were free to decide whether they would like to participate or not. Those who finally decided to participate signed an informed consent form.

After their collection, all questionnaires were locked in a secure place in the CSSEN office, and similarly, all electronic data were downloaded from the platform and saved in a password-protected excel file. Afterwards, all hard-copy questionnaires, as well as the electronic data, were coded on an SPSS file and then destroyed and erased from the platform. The electronic SPSS file of the data was secured on a password-protected USB, which was only accessible to the researchers.

### 2.4. Statistical Analysis

For the statistical analysis, we used the IBM SPSS version 23. As the first step and before testing our research questions, we performed normality tests using the Kolmogorov–Smirnov test and we also used histograms, Q–Q plots and skewness and kurtosis to check normality. Results indicated that our data were not normally distributed. Afterwards, we tested the CAQ internal consistency using Cronbach’s alpha, and we also ran preliminary analyses for the descriptive statistics of the study. Finally, to test our research questions, we ran non-parametric tests (i.e., Spearman rho, Mann–Whitney test and Kruskal–Wallis test).

## 3. Results

### 3.1. Descriptive Statistics

Preliminary analyses were conducted for all the demographic characteristics of the sample. The demographic characteristics of the sample by group are presented in Table S1.

Additionally, the specific demographic characteristics for SWD are presented in Table S2.

### 3.2. Inferential Statistics

#### 3.2.1. Differences in GPA and Adaptation between Students with and without Disabilities

A Mann–Whitney U test was performed to compare the two groups’ differences in GPA and adaptation. Results indicated significant differences in GPA, *z* = 4.47, *p* = 0.001, with students without disabilities having a higher GPA (Mdn = 148.10) than SWD (Mdn = 106.90). Differences were also found in adaptation, *z* = 10.14, *p* = 0.001, with students without disabilities having higher adaptation scores (Mdn = 174.23) than SWD (Mdn = 80.77). Table S3 shows these results.

#### 3.2.2. Differences between SWD in GPA and Adaptation According to (a) Their Type of Disability and (b) the Existence of Disability Comorbidity

(a) A Kruskal–Wallis H test was performed to compare differences in GPA and adaptation according to the type of disability. A statistically significant difference was found between the mean ranks of at least one pair of the categories of disability only for GPA, H(5, 127) = 13.73, *p* = 0.017 with a mean rank 114.75 for mobility disability, 78.79 for health problems, 72.82 for auditory disability, 70.56 for mental health problems, 63.50 for ADHD and 56.90 for learning disabilities. The post hoc comparisons showed significant differences in GPA only between the pair of learning and mobility disabilities (*p* = 0.032, adjusted using the Bonferroni correction). No significant differences were found for adaptation, H(5, 127) = 4.61, ns. Table S4 shows these results;
(b) A Mann–Whitney U test was performed to compare differences in GPA and adaptation according to the existence of disability comorbidity. Statistically significant differences were found only for GPA *z* = 2.126, *p* = 0.034, with SWD without comorbidity having a higher GPA (Mdn = 66.91) compared to those with a comorbidity (Mdn = 47.45). No significant differences were found for adaptation, *z* = 1.756, ns. Table S5 shows these results.

#### 3.2.3. SWD’s Differences in GPA and Adaptation According to the Type of Academic Accommodations

From the Mann–Whitney U test, no significant differences were found for GPA *z* = 1.144, ns or adaptation GPA *z* = 0.568, ns according to the type of teaching accommodations, and Table S6 shows these results. However, the Kruskal–Wallis H test showed statistically significant differences between the mean ranks of at least one pair of the exam accommodations only for GPA H(2, 127) = 8.045, *p* = 0.018 with a mean rank 81.06 for special exam accommodations, 71.55 for combination of exam accommodations and 54.97 for basic accommodations. The post hoc comparisons showed significant differences only between the pair of basic and combination exam accommodations (*p* = 0.044, adjusted using the Bonferroni correction). No significant differences were found for adaptation H(2, 127) = 1.737, ns. Table S7 shows these results.

#### 3.2.4. SWD’s Differences in GPA and Adaptation According to the Existence of Therapy

From the Kruskal–Wallis H test, no statistically significant differences were observed on GPA H(2, 127) = 3.409, ns., or on adaptation H(2, 127) = 1.517, ns. between SWD who were in therapy, those who had therapy in the past and those have never undergone therapy. Table S8 shows these results.

#### 3.2.5. Differences in SWD’s GPA and Adaptation Scores According to Their Perceptions of Receiving a Specialised Package of Counselling Psychology Interventions

The Mann–Whitney U test indicated statistically significant differences only for adaptation *z* = −2.125, *p* = 0.034, with SWD who were positive that extra support could enhance their academic experience having higher adaptation scores (Mdn = 69.41) than SWD who were negative (Mdn = 55.10). No differences were found for GPA *z* = −0.124, ns. Table S9 shows these results.

## 4. Discussion

The main goal of this study was to examine the differences in adaptation and academic performance between university students with and without disabilities and to understand the role of the support provided to SWD. The main findings of the study showed that students with and without disabilities have significant differences in GPA and adaptation. Furthermore, we found that SWD only have differences in GPA according to the type of their disability, the existence of comorbidity and exam accommodations. Additionally, no differences in GPA and adaptation were found between SWD users and non-users of psychological therapy. Finally, it was revealed that there is some prospect to consider an additional approach in supporting SWD by implementing interventions from a counselling psychology perspective.

### 4.1. Differences between Students with and without Disabilities

Beginning with our first question, our findings about differences in academic adaptation and performance between students with and without disabilities are in accordance with previous studies [17,18,19]. Our result indicates that SWD have a lower GPA and feel less satisfied with the university, their course and their socialisation at the university. This may be because of the additional challenges caused by their disability and other factors associated with disability. SWD face several challenges not only because of the obstacles posed by their disability but also with the associated distress and the emotional burden of the involved stigma, which seems to play a negative role in their adaptation to the university [48]. These difficulties also seem to affect their academic performance [13,14]. Of course, not all SWD face the same or even similar difficulties [81], but the general inference is that compared to students without disabilities, they face more difficulties in adaptation and academic performance.

### 4.2. Differences between SWD in GPA and Adaptation

#### 4.2.1. Disability Type and Comorbidity

Concerning our second question, our findings revealed differences between SWD concerning GPA but not adaptation. More specifically, we found significant differences in SWD’s GPA according to the type of disability, with mobility disability having a higher GPA, followed by health problems, auditory disability, mental health problems, ADHD and learning disabilities. From the disability types, a statistical significance was observed only between students with learning disabilities and mobility disabilities, with the latter having a higher GPA. This might be because students with mobility disabilities mainly face obvious and immediately discernible barriers in accessibility which do not directly impact their GPA [82]. Also, students with mobility disabilities do not face similar problems as students with learning disabilities, such as memory, processing, perceptual and comprehension difficulties, which require support such as specialised pedagogical approaches and adjustment of learning materials [82]. Contrary to our result, the study of Dong and Loucas [43] reported that students with cognitive and mental disabilities perform better than other disability groups, but this applied only in the third semester of the study. This discrepancy in findings suggests that the impact of disabilities on academic performance may vary depending on the specific disability type and the stage of academic progression and that further research is needed to explore and comprehend these differences among students with various disabilities.

Another finding from our second question was that students with disability comorbidity had a lower GPA than students who only had a single disability, and this finding was consistent with other studies (e.g., [3,83,84,85]). Having multiple disabilities can lead to more complex challenges, which can intensify the adversities faced by each student. For example, it was found that the coexistence of ADHD and learning disabilities [86] affected cognition, processing speed, memory, attention, or other skills important for academic success, potentially leading to lower grades. Furthermore, students with learning disabilities, dyslexia and mobility disabilities may experience social isolation, stigma and stress, which may affect their emotional wellbeing and, indirectly, their academic performance [87,88]. The result of a lower GPA in students with comorbidities highlights the importance of individualising the available support by offering tailored and adaptive strategies which will allow students to progress successfully in their academic careers [89,90].

Finally, for our second question, we did not find any differences in adaptation. The lack of significant differences in adaptation indicates that SWD in our sample show more or less similar levels of university adaptation regardless of their disability type and the existence of comorbidity. This is contrary to the study which found that mental disabilities and ADHD had significantly lower adaptation from other disability types [18]. Given that in our sample, SWD’s adaptation is considered low, it is possible that students may have adopted similar unhelpful coping strategies due to self-stigmatisation or community expectations that having a disability is undeniably accompanied by problems in university adjustment. Moreover, this finding may also be attributed to the fact that, generally, SWD may procrastinate seeking support for several reasons [91]. Unfortunately, all the available findings from the limited existing studies comparable to ours have not primarily focused on adaptation differences between SWD and hence, our results must be interpreted as preliminary and with caution.

#### 4.2.2. Academic Accommodations

SWD must be provided with all the necessary tools to be accommodated through their studies [41]. To achieve this, universities must be able to assess their needs and provide them with the necessary support to assist them with their difficulties. One method of supporting SWD is the provision of academic accommodations, and our third research question explored differences in GPA and adaptation according to the type of provided accommodations. From the results, no differences were found in GPA or adaptation according to the type of teaching accommodations, but we found differences in GPA according to the type of exam accommodations. SWD who received the basic teaching accommodations (e.g., extension in submitting assignments, simplification of assignment instructions and outcomes and exemption of spelling, punctuation, syntax and grammar errors in assignments) had similar scores in GPA and adaptation with SWD who received a combination of basic and other teaching accommodations (e.g., frequent breaks during lectures, provision of copies of the lecture notes and audio-recording of lectures). This indicates that the two types of teaching accommodations work similarly for SWD in our sample. Perhaps the absence of such differences shows that teaching accommodations allow access to equal educational opportunities in class and not necessarily achievement of higher grades or better adaptation between SWD. At the same time, it is important to acknowledge that our finding may be attributed to the uneven number of students in each teaching accommodation group (101 in the basic accommodations and 26 in the combination) and also in each disability group. Generally, as we have seen in the literature review, past studies have primarily focused on the join impact of teaching and exam accommodations on GPA and adaptation and not separately, making it difficult to compare our findings. Therefore, our result must be interpreted as preliminary and definitely more research is needed to further explore and understand this.

Significant differences in SWD’s GPA were found according to the type of exam accommodations. Specifically, differences were observed between students who had a combination of exam accommodations and students who only had basic exam accommodations. This means that SWD who had a combination of exam accommodations (i.e., oral examination, frequent breaks during the examination, private examination in the absence of other students, use of computers to type answers, and use of a scribe) gained better grades than those who only had basic exam accommodations such as additional examination time, simplification of the exam, explanation of unknown words and exemption of spelling, punctuation, syntax and grammar errors. The differences in our findings may be because SWD who receive a combination of exam accommodations have very specific difficulties, and the accommodations are tailored to their needs. This suggests that individualising and differentiating exam accommodations may have a positive impact on the academic performance of SWD. Individualised and structured exam accommodations, as well as combining exam methodologies, appear to provide students with the ability to build on their strengths and mitigate the challenges associated with their disability. This can lead to better academic performance, demonstrating the value of carefully designed and personalised exam accommodations [92].

#### 4.2.3. Psychological Therapy

Interestingly, for our fourth question, we found a non-significant difference in GPA and adaptation between SWD who received psychological therapy and those who did not. Specifically, students who were in therapy during data collection, students with past experience of therapy and non-users of therapy had similar GPA and adaptation scores. On the one hand, our result could possibly be interpreted based on the fact that SWD may not seek therapy for the same reasons and may not enter therapy merely to increase their GPA nor to work directly on any transition or adaptation issues. Some other reasons for seeking therapy while studying at university could vary from serious mental health issues, loss and crisis support, to anxiety, relationship issues, academic-related distress, self-esteem and even financial issues [37,61]. All these reasons for entering therapy may not necessarily or directly affect SWD’s GPA and adaptation. Furthermore, as some studies suggest, SWD may not seek support from therapy and instead may use other means of support that could possibly help them with their GPA and adaptation [32,35,36].

On the other hand, our result was not unexpected based on previous findings concerning SWD’s gains from therapy. More specifically, existing studies found that SWD, compared to their peers without disabilities, demonstrated significantly fewer reduction of psychological and academic distress, were more likely to drop-out from therapy and also be referred from campus services to external services [71,72]. Although therapy may potentially be psychologically supportive for SWD, their disability, but also the environmental and personal problems related to their disability, can block a number of SWD from internalising the benefits of therapy. It is evident from past studies that even though SWD who seek treatment appear to have similar levels of distress in some areas (i.e., depression, eating concerns, hostility, family distress and social anxiety), they often struggle with more complex difficulties compared to their peers without disabilities and they present with greater academic concerns, general anxiety and also higher rates of self-harming behaviours, suicidal ideation and attempts [93,94]. Furthermore, a recent study with a large sample size (SWD = 6382 and students without disabilities = 86,966) reported that SWD have a higher prevalence rate of depression, anxiety, self-harming behaviours and suicide than students without disabilities [95]. Therefore, perhaps it is harder for some of them to transform therapy into a positive experience which can enhance their academic performance and their adaptation, and this calls for a different perspective in providing psychological support services to SWD.

Evidence of poorer therapeutic outcomes for individuals with disabilities, when compared to individuals without disabilities, can also be found in large national health systems (e.g., in the UK) where evidence-based psychological therapies are offered [96]. At the same time, there is an ongoing debate about what works best for individuals with disabilities, with evolving debates on the topics of therapy effectiveness, suitability of psychotherapeutic modalities, therapists’ expertise in disability and disability-oriented interventions [97,98,99]. Having said this, we do not know exactly our participants’ therapy characteristics. For example, we do not know if they engaged in individual or group therapy, brief or long-term therapy, counselling or psychotherapy, which therapeutic modality-approach was followed, the reasons for embarking in therapy, and, more importantly, if their therapy was oriented according to the underlying basis of their disability. If we knew this information, then the interpretation of our results might have been different, especially as the literature points out that knowledge of therapy factors can be crucial in comprehending therapy effectiveness for SWD [31]. Nevertheless, our finding suggests that SWD with or without therapy have similar academic performance and adaptation levels and perhaps the existence of therapy can be beneficial only if adapted to become clinically relevant and responsive to their needs.

#### 4.2.4. Counselling Psychology

For our last question on SWD’s perceptions of a proposed specialised package of counselling psychology interventions, which consists of disability psychoeducation, mindfulness, social-emotional learning techniques, working with acceptance, self-stigma, attributional styles and self-determination, we found that SWD who were positive that this package could enhance their academic experience had significantly higher adaptation scores from SWD with negative opinions. Students who are motivated to receive extra support may be more prone to accept help and benefit from this intervention. Our finding can be partly explained by previous studies highlighting the relation of self-stigma with negative attitudes towards psychotherapy [100] and, more specifically, SWD’s perceptions of stigma, which can prevent them from requesting support [94,101]. Furthermore, our findings can be interpreted by the notion of help-seeking avoidance in university students [102]. Even though this notion has mostly been researched in the general population of university students, it may also apply to SWD. Help-seeking avoidance consists of psychological beliefs and other behaviours which prevent a student from requesting psychological help when needed. Wilson and Deane [103] have broadly categorised these barriers in help-seeking as person-related (e.g., self-esteem, fear and embarrassment of using a service) and treatment-related (e.g., therapist competency, uncertainty of therapy effectiveness, waiting lists). Therefore, we consider that the exploratory finding of our study may be promising in directing our effort to support SWD towards the creation of a supplementary approach, which will be adapted to their actual needs and minimise such barriers to receiving help.

Searching the literature on the improvements of access to treatment and on adaptations of psychological therapies for individuals with intellectual disabilities [104], we have realised that although research on this topic is steadily growing, such interventions are not fully applicable to all disability types we encounter for students in HE. This is exactly the reason why university counselling centres need to be proactive and design new interventions or adapt existing ones to SWD’s needs, and we believe that counselling psychology can significantly contribute to this endeavour.

As reported in the literature, counselling psychologists work in a variety of settings, but one constant employment is within university counselling services [105,106,107,108]. Counselling psychology was historically established in university counselling centres, with E.G Williamson creating a counselling approach which focused on helping students who faced academic, financial and socioemotional problems, including students with disabilities, to adapt to university [109]. Furthermore, the relationship between counselling psychology and disability was also deeply-rooted in history. As a matter of fact, the developmental trajectory of counselling psychology as a distinct applied discipline in the US was influenced, among other factors, by the need to provide support services to disabled veterans of the second world war [110]. Thus, it was expected that a considerable number of studies would exist to describe the work of counselling psychologists with individuals with disabilities and, more specifically, with SWD in HE, but surprisingly, this was not the case. In her theoretical paper, Jones [73] reported that historically, the most commonly used therapies for the treatment of individuals with disabilities were psychopharmacology and applied behavioural models and that very little was known about counselling psychologists’ work with this population. Finally, the study of Kanelaki and Kanelakis [106] revealed that although counselling psychologists engage with disability in their practice, this engagement was generic and much less than what was historically expected.

Nevertheless, while it is understood that counselling psychology may not have fully embraced clinical work and research in the area of disability, we strongly believe that this can change in the future. Counselling psychology is a discipline that views disability as a psychosocial construct [111] and values diversity through pluralistic practices [112,113]. Hence, this approach can lead to a deeper understanding of SWD’s phenomenology in order to create and implement new interventions to strengthen university transition and adaptation. One of the most powerful therapeutic elements in counselling psychology, which can be utilised to achieve this goal, is the use of the therapeutic relationship [114], and there is a valid reason that we place the therapeutic relationship (TR) at the heart of applying psychological interventions. As a matter of fact, a respectable number of studies show TR to be one of the most reliable predictors of outcomes in many therapeutic approaches, including the discipline of counselling psychology [115,116,117]. Although it might be out of the scope of this paper to analyse how specifically TR can be utilised in the proposed package of interventions, we briefly present a basic theoretical proposition that is grounded in research and which could help us understand its association with our result. So, based on previous research on the valuable contributions of the therapeutic relationship with individuals with disabilities, we identified a number of factors that must be taken into account when applying a newly proposed package of counselling psychology interventions. For example, a number of papers [73,118,119] suggest that some individuals with disabilities may present with limited experience of relationships and social networks, that they may feel as if they are in a less powerful position in relationships and hence that may be less confident and expressive in their sessions. Additionally, it is suggested that some therapists may take an authoritarian or patronising stance and even be biased towards their clients’ disabilities. Even though we understand that this may not be the norm for all therapists and all young adults with disabilities, the point is to acknowledge how such characteristics could hinder the implementation of an intervention. By working in and on the TR, therapists can become more attentive to how their own characteristics, but also their clients’ experiences of other relationships, may be interplayed in session in the form of transference and countertransference while applying interventions. Therefore, we hypothesise that the element of therapeutic relationship can have a significant potential in the effective application of such interventions. As this is only a tentative hypothesis, it provides us the opportunity to proceed with new research of this nature.

## 5. Limitations

While the findings of the current study have important implications for the support of SWD, they should be understood in the context of several limitations. Firstly, the data were collected from a single university in Cyprus and cannot be assumed to be applicable to all university students. Additionally, the questionnaires were completed only in Greek, and a significant number of international students were excluded from participating. Secondly, generalisations cannot be applied to SWD who have not registered with CSSEN. In addition, some SWD may have been hesitant to disclose their disability to receive accommodations, and therefore, they may have used other support means, something which was overlooked in the current study. Thirdly, data were collected during one semester, and this did not allow for measurements at different times. Also, the period of data collection must be considered transitional due to the COVID-19 pandemic since students had fully returned to campus without any restrictions a semester prior to data collection. Fourthly, the findings were derived from self-report questionnaires, which are based on subjective perceptions. Moreover, although the questionnaire utilised for adaptation had good reliability and was easy and fast to use, it evaluated adaptation as unidimensional, in contrast with most previous studies that used questionnaires that perceived adaptation as multidimensional. A fifth limitation might be the data which were not collected and which could have influenced our findings, such as therapy characteristics, disability severity, other resources of support that SWD could use instead of accommodations, studying in part-time or full-time mode, having an equal number of participants on each disability category, year and area of study. Finally, a sixth limitation might have been the dual role of researchers who were also members of the CSSN. Two of the three members were actively involved in the procedure of assessment and provision of academic accommodation to SWD in the university. Therefore, it is possible that impartiality may not have been fully succeeded specifically for the sample of SWD. However, we believe that this may have been minimised for two reasons: firstly, anonymity in data collection was strictly preserved, and secondly, data analysis was carried out by a third researcher who had very limited involvement in the assessment process and identification of SWD.

## 6. Suggestions for Further Research

Based on our findings, a number of recommendations for future research could be provided. Future research needs to address issues concerning the effectiveness of the support provided to SWD. Therefore, more exploratory studies could be developed to empirically investigate the influence of academic accommodations, psychological therapy and other means of support on several aspects of SWD’s experience. In addition, perhaps a longitudinal design would allow for measurements at different times during students’ education, as well as after graduation. This would inform our understanding of the challenges faced by SWD, and it could also help us draw inferences for the long-term efficiency of the support provided to SWD during their studies. Moreover, future studies may wish to address other variables and also explore more complex relationships between disability, support and students’ personal (e.g., stigma, personality) and environmental factors (e.g., family support). Additionally, we have seen that SWD are not a homogenous group; they may face different difficulties and have different approaches and reactions in receiving support. Therefore, ensuring a sufficient sample size for comparison across disability types is another line of research that could be opened based on our results. Finally, as we have stressed the importance of the association between the philosophy of counselling psychology and disability, further research could be very beneficial for this specific literature.

## 7. Conclusions and Implications for Practice

In conclusion, our study revealed that SWD differ significantly from the general student population in their adaptation to university as well as in their academic performance. Our results highlight the need for a continuous provision of specialised support to facilitate SWD’s smooth trajectory in HE. This support could be provided after careful assessment of the students’ disability type, and reasonable adjustments, with special emphasis on exam accommodations, should be provided. Additionally, it seems that extra attention should be given to the provision of psychological therapy due to the fact that each SWD has unique difficulties, which are not always tackled by traditional psychological interventions. Therefore, we believe that counselling psychologists, with their pluralistic practices, can value diversity by creating a disability-oriented support system in university counselling centres, where interventions will be designed and tailored to match SWD’s needs.

In light of our study’s findings, some practical implications could be suggested to inform the practice of professionals who work with SWD. Our study confirmed past studies and indicated that SWD are indeed considered academically at risk, compared to their peers, as they have lower academic performance and more adaptation difficulties. As universities generally strive to promote academic performance, and since one of their core missions is excellence in teaching and research, there is a need to proceed with more empirical research to find ways to improve academic performance specifically for SWD. Furthermore, as adaptation difficulties are more prevalent in SWD compared to their peers without disabilities, it is understood that universities should focus on tackling transitional issues. For many SWD, this transition means that they will probably need to make independent decisions in disclosing their disability as well as requesting and applying support aids in their new academic environment. Hence, creating the necessary conditions to better evaluate students’ needs and, more specifically, to identify a disability might be one important action required by universities. To achieve this, universities should be aware of the barriers that SWD may face and create a safe environment to help them process the fear of stigmatisation, which might prevent them from declaring their disability and receiving support services. Moreover, due to the fact that the transition to university coincides with the developmental phase of separation and individuation, university support services should consider ways to help SWD negotiate issues of identity and self-esteem and also strengthen their psychological resilience to be able to cope with adaptation issues.

The careful assessment of SWD should be another critical aim for their academic support, as we have seen that SWD have differences in GPA according to their disability. Not all SWD necessarily struggle with academic performance issues, but the difficulties caused by the type of their disability seem to affect their GPA. This is also true for students who present with the issue of comorbidity. Having more than one disability can burden a student and lead to underperformance. Therefore, student disability offices and counselling centres must carefully assess students’ history and find any existing diagnosis-gaps between high school and HE. The conventional view of disability as a persistent and unchanging limitation may actually hinder professionals’ assessment methods and miss the real needs of SWD. Hence, perhaps adjusting assessment methods towards an understanding of the dynamic aspect of disability [120] can actually assist professionals in better assessing the developmental changes in disability, which can influence SWD academic difficulties.

It is evident that not all support measures taken by universities can be effective and that the need to identify SWD’s actual needs is essential for personalised support. Based on our findings, teaching and exam academic accommodations have their own special role in SWD’s performance. Therefore, better monitoring of SWD’s academic progress and use of the provided academic accommodations is another mechanism which can be put in place. Disability offices, counselling centres, academic advisors and faculty should work together to achieve a better monitoring of the effectiveness of the provided accommodations but also to overcome the barriers in implementing accommodations that are often reported in the literature [121].

Our study has the potential to inform professionals who work therapeutically with SWD and, more specifically, university counselling centres. As we have seen in other studies, psychological therapy is generally effective for the general student population, but there is still not much evidence to confirm or reject the hypothesis that it can be effective for SWD’s academic performance and adaptation. Our study’s results revealed that SWD users and non-users of therapy had similar GPA and adaptation scores. Therefore, careful consideration should be given to what works best for whom. A pluralistic evaluation of SWD’s needs is far more important than assuming that SWD’s concerns are only disability-related or that psychological therapy might be the only means of help. We still believe that psychological therapy can assist SWD, but only if adapted to become clinically relevant and responsive to their needs, while at the same time, therapists should be able to address obstacles to treatment effectiveness.

Moreover, based on our result of the SWD’s positive perceptions in receiving extra support, other resources should be utilised too. Specifically, we explored if a package of interventions designed by counselling psychologists consisting of disability psychoeducation, mindfulness, social-emotional learning techniques, working with acceptance, self-stigma, attributional styles and self-determination could seem appealing to SWD in terms of helping with their academic experience. Of course, as this package of interventions is not yet applied, we cannot reveal much more or discuss its efficiency. Nevertheless, the intention here is to actually cultivate the idea of exploring other means of support and to encourage all those involved in HE to find ways to help SWD have a smoother university adaptation and academic performance. From past research, it seems that psychoeducation, social support, orientation programs and peer-support networking are some means of support, among many others, which can actually help SWD. If universities want to offer the best possible options for supporting their students, then the most recent evidence-based research should be followed and adequate resources and funding should be allocated.

## Data Availability

The data of this study are unavailable due to privacy and ethical restrictions.

## Supplementary Materials

The following supporting information can be downloaded at: https://www.mdpi.com/article/10.3390/bs13100862/s1, Table S1: Demographic characteristics of the sample by group; Table S2: Disability group demographic information. Table S3: Mann–Whitney U tests for differences in GPA and adaptation between students with and without disabilities (significance level *p* < 0.05). Table S4: Kruskal–Wallis H test for differences between SWD in GPA and adaptation according to the type of disability (significance level *p* < 0.05). Table S5: Mann–Whitney U tests for differences between SWD in GPA and adaptation according to disability comorbidity (significance level *p* < 0.05). Table S6: Mann–Whitney U tests for differences between SWD in GPA and adaptation according to the type of teaching academic accommodations (significance level *p* < 0.05). Table S7: Kruskal–Wallis H test for differences between SWD in GPA and adaptation according to the type of exam accommodations (significance level *p* < 0.05). Table S8: Kruskal–Wallis H test for differences between SWD in GPA and adaptation according to the existence of therapy (significance level *p* < 0.05). Table S9: Mann–Whitney U tests for between SWD in GPA and adaptation according to their perceptions of a specialised package of counselling psychology interventions (significance level *p* < 0.05).
