# Supplementary material for: Adaptation, Academic Performance and Support: Students with and without Disabilities and Future Considerations for Counselling Psychology
Source: Behav Sci (Basel). 2023 Oct 20;13(10):862. doi: 10.3390/bs13100862 (PMC10604788; doi:10.3390/bs13100862)
Supplement: Supplementary file 1 [file behavsci-13-00862-s001.zip › behavsci-2651091-supplementary.pdf]

Table S1. Demographic characteristics of the sample by group.

Demographic Variable	Disability (*n* = 127)	Non-Disability (*n* = 127)	Total (*n* = 254)
Gender:			
Male	43.3% (*n* = 55)	44.9% (*n* = 57)	44.1% (*n* = 112)
Female	56.7% (*n* = 72)	55.1% (*n* = 70)	55.9% (*n* = 142)
Nationality:			
Cypriots	55.9% (*n* = 71)	45.7% (*n* = 58)	50.8% (*n* = 129)
Greeks	44.1% (*n* = 56)	54.3% (*n* = 69)	49.2% (*n* = 125)
Residence Status:			
Living Alone	38.6% (*n* = 49)	55.1% (*n* = 70)	46.9% (*n* = 119)
Living with Partner	13.4% (*n* = 17)	7.1% (*n* = 9)	10.2% (*n* = 26)
Living with Parents	43.3% (*n* = 55)	23.6% (*n* = 30)	33.5% (*n* = 85)
Living with Roommates	4.7% (*n* = 6)	14.2% (*n* = 18)	9.4% (*n* = 24)
Level of Study:			
Bachelor	81.9% (*n* = 104)	89.8% (*n* = 114)	85.8% (*n* = 218)
Master	16.5% (*n* = 21)	10.2% (*n* = 13)	13.4% (*n* = 34)
Doctorate	1.6% (*n* = 2)	0	0.8% (*n* = 2)
Program of Study:			
Medicine	16.5% (*n* = 21)	9.4% (*n* = 12)	13% (*n* = 33)
Sciences	18.9% (*n* = 24)	28.3% (*n* = 36)	23.6% (*n* = 60)
Social Sciences and Humanities	33.1% (*n* = 42)	40.9% (*n* = 52)	37% (*n* = 94)
Education	7.9% (*n* = 10)	3.1% (*n* = 4)	5.5% (*n* = 14)
Business	11% (*n* = 14)	11.8% (*n* = 15)	11.4% (*n* = 29)
Law	12.6% (*n* = 16)	6.3% (*n* = 8)	9.4% (*n* = 24)
Year of Study:			
First	26.8% (*n* = 34)	26.8% (*n* = 34)	26.8% (*n* = 68)
Second	23.6% (*n* = 30)	24.4% (*n* = 31)	24% (*n* = 61)
Third	20.5% (*n* = 26)	18.1% (*n* = 23)	19.3% (*n* = 49)
Fourth	13.4% (*n* = 17)	24.4% (*n* = 31)	18.9% (*n* = 48)
Fifth and above	15.7% (*n* = 20)	6.3% (*n* = 8)	11% (*n* = 28)
Mean Age	21.34 (*SD* = 1.97)	20.69 (*SD* = 1.78)	21.02 (*SD* = 1.90)
Mean Grade Point Average	2.65 (*SD* = 0.91)	3.14 (*SD* = 0.64)	2.89 (*SD* = 0.82)
Mean University Adaptation	64.65 (*SD* = 9.99)	87.50 (*SD* = 18.17)	76.08 (*SD* = 18.58)

Table S2. Disability group demographic information.

**Demographic Variable**	**SWD (n = 127)**
Disability Type:	
Learning	62.2% (n = 79)
ADHD	7.9% (n = 10)
Auditory	8.7% (n = 11)
Mobility	3.1% (n = 4)
Health Problems	11% (n = 14)
Mental Health Problems	7.1% (n = 9)
Comorbidity:	
Yes	15% (n = 19)
No	85% (n = 108)
Psychological Therapy:	
Yes, in the past	29.9% (n = 38)
Yes, currently	22.8% (n = 29)
Never	47.2% (n = 60)
Mode of Study:	
Conventional Study	81.1% (n = 103)
Distance Learning	18.9% (n = 24)
Teaching Accommodations:	
Basic	79.5% (n = 101)
Combination	20.5% (n = 26)
Exams Accommodations:	
Basic	49.6% (n = 63)
Special	7.1% (n = 9)
Combination	43.3% (n = 55)
Beliefs about Extra Specialized Package:	
Positive	62.2% (n = 79)
Negative	37.8% (n = 48)

Table S3. Mann–Whitney U tests for differences in GPA and adaptation between students with and without disabilities (significance level $p < 0.05$).

		GPA				Adaptation			
**Independent Variable**	**N**	**Median**	**U**	**Z**	**Sig**	**Median**	**U**	**Z**	**Sig**
Students without Disabilities	127	148.10				174.23			
			10.681	4.472	0.001		13.999	10.140	0.001
Students with Disabilities	127	106.90				80.77			

Table S4. Kruskal–Wallis H test for differences between SWD in GPA and adaptation according to the type of disability (significance level $p < 0.05$).

		GPA				Adaptation			
**Independent Variable**	**N**	**Mean Rank**	**H**	**df**	**Sig**	**Mean Rank**	**H**	**df**	**Sig**
Learning Disabilities	79	56.90	13.727	5	0.017	61.64	4.610	5	0.465
ADHD	10	63.50				61.55			
Auditory Disabilities	11	72.82				54.41			
Mobility Disabilities	4	114.75				79.00			
Health Problems	14	78.79				77.75			
Mental Health Problems	9	70.56				74.61			

Table S5. Mann–Whitney U tests for differences between SWD in GPA and adaptation according to disability comorbidity (significance level $p < 0.05$).

		GPA				Adaptation			
**Independent Variable**	**N**	**Median**	**U**	**Z**	**Sig**	**Median**	**U**	**Z**	**Sig**
SWD with Comorbidity	19	47.45				50.34			
			1.340	2.126	0.034		1.285	1.756	0.079
SWD without Comorbidity	108	66.91				66.40			

Table S6. Mann–Whitney U tests for differences between SWD in GPA and adaptation according to the type of teaching academic accommodations (significance level $p < 0.05$).

		GPA				Adaptation			
**Independent Variable**	**N**	**Median**	**U**	**Z**	**Sig**	**Median**	**U**	**Z**	**Sig**
Basic	101	62.10				63.06			
			1.504	1.144	0.252		1.408	0.568	0.570
Combination	26	71.37				67.65			

Table S7. Kruskal–Wallis H test for differences between SWD in GPA and adaptation according to the type of exam accommodations (significance level $p < 0.05$).

		GPA				Adaptation			
**Independent Variable**	**N**	**Mean Rank**	**H**	**df**	**Sig**	**Mean Rank**	**H**	**df**	**Sig**
Basic	63	54.97	8.045	2	0.018	59.88	1.737	2	0.420
Special	9	81.06				63.39			
Combination	55	71.55				68.82			

Table S8. Kruskal–Wallis H test for differences between SWD in GPA and adaptation according to the existence of therapy (significance level $p < 0.05$).

		GPA				Adaptation			
**Independent Variable**	**N**	**Mean Rank**	**H**	**df**	**Sig**	**Mean Rank**	**H**	**df**	**Sig**
Past Therapy	38	60.28	3.409	2	0.182	62.92	1.517	2	0.468
Present Therapy	29	75.07				71.26			
Non-users of Therapy	60	61.01				61.18			

Table S9. Mann–Whitney U tests for differences between SWD in GPA and adaptation according to their perceptions of a specialized package of counselling psychology interventions (significance level $p < 0.05$).

		GPA				Adaptation		
**Independent Variable**	**N**	**Median**	**U**	**Z**	**Sig**	**Median**	**U**	**Z**	**Sig**
Positive Perceptions	79	64.32				69.41			
			1.871	−0.124	0.901		1.469	−2.125	0.034
Negative Perceptions	48	63.48				55.10			
